# Supplementary material for: Genetic dissection of Nodal and Bmp signalling requirements during primordial germ cell development in mouse
Source: Nat Commun. 2019 Mar 6;10:1089. doi: 10.1038/s41467-019-09052-w (PMC6403387; doi:10.1038/s41467-019-09052-w)
Supplement: Supplementary file 1 — Supplementary Information [file 41467_2019_9052_MOESM1_ESM.pdf]

Supplementary Information

Genetic dissection of Nodal and Bmp signalling requirements during primordial germ cell development in mouse

Anna D. Senft, Elizabeth K. Bikoff, Elizabeth J. Robertson and Ita Costello

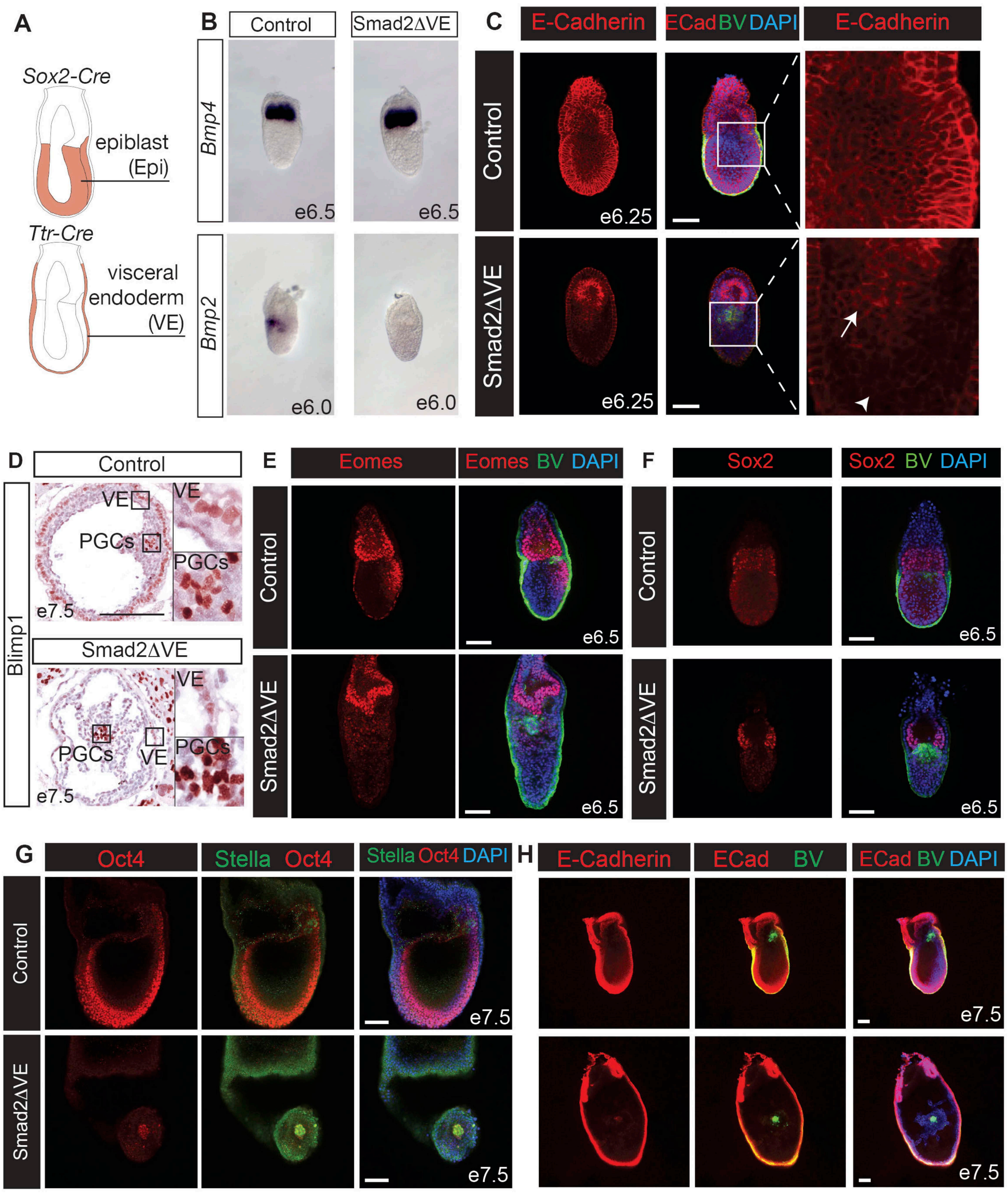

Supplementary figure 1: **Conditional Smad2 inactivation in the visceral endoderm results in expansion of the PGC niche.**

(A) Tissue specific activity of the Sox2 and Ttr Cre deleter strains. (B) Whole-mount *in situ* hybridisation analysis of *Bmp4* and *Bmp2* expression in e6.5 and e6.0 control and Smad2ΔVE embryos. (C) E-Cadherin (ECad) staining of e6.25 control and Smad2ΔVE BV^{+} embryos. Arrow indicates E-Cadherin staining in BV-expressing cells. Arrowhead indicates lack of E-Cadherin staining in surrounding mesoderm-like cells. (D) Immunohistochemistry (IHC) staining of sections of e7.5 control and Smad2ΔVE embryos using a Blimp1 antibody. Boxed regions are expanded on the right. Scale bar = 200μm. (E) Eomes expression in control and Smad2ΔVE BV^{+} e6.5 embryos. (F) Sox2 expression in BV^{+} e6.5 embryos. (G) Stella and Oct4 co-localization in e7.5 control and Smad2ΔVE embryos. (H) E-Cadherin staining of e7.5 control and Smad2ΔVE embryos. All IF images are counterstained with DAPI. All IF scale bars = 100μm.

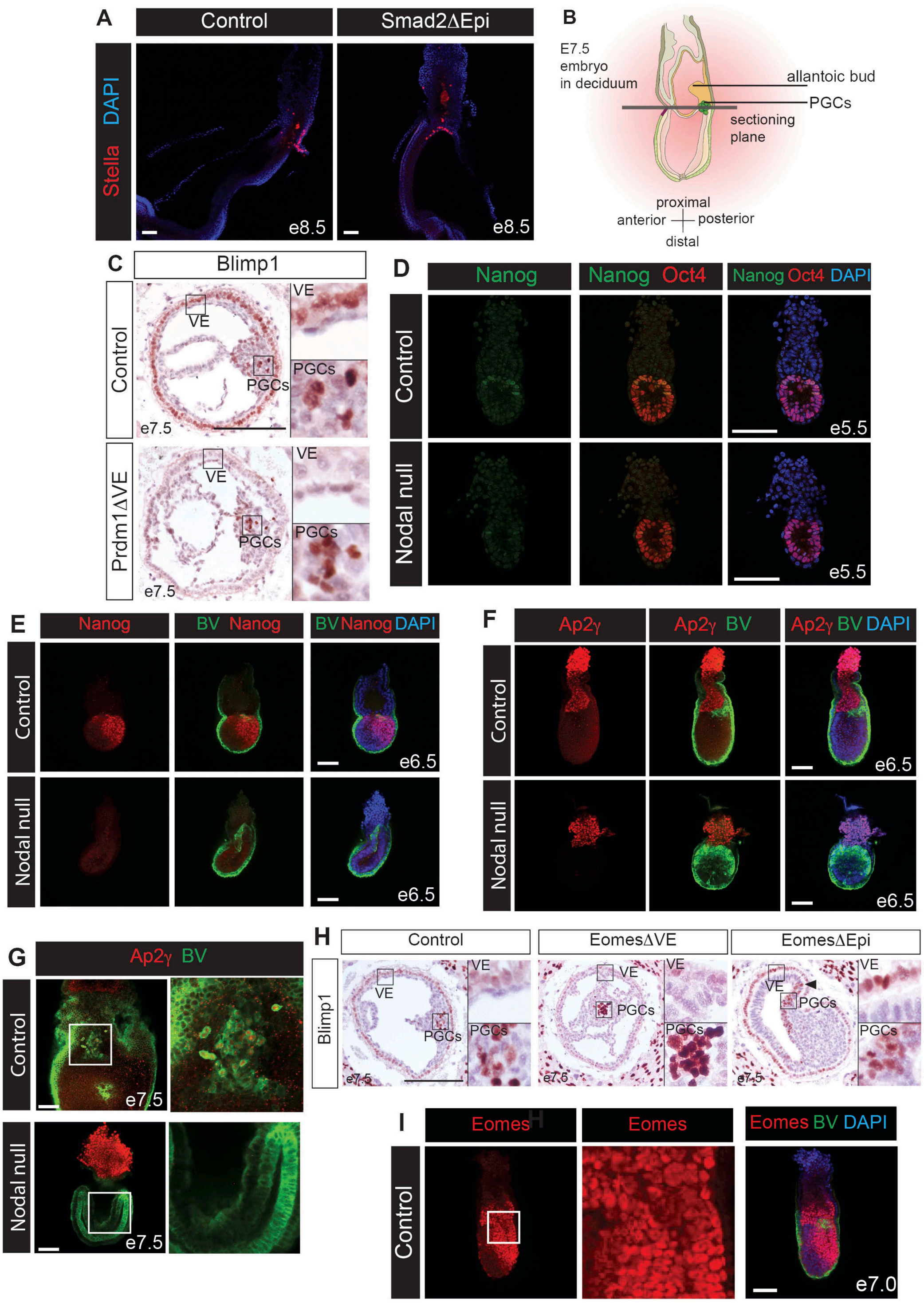

Supplementary figure 2: **Smad2, Prdm1, Nodal and Eomes functional contributions during PGC development.**
(A) Stella IF staining in control and Smad2ΔEpi e8.5 embryos. (B) Plane of section used for IHC staining. (C) Blimp1 IHC in control and Prdm1ΔVE sections shows selective deletion of Blimp1 in the VE of Prdm1ΔVE embryos fails to prevent PGC formation. Scale bar = 200μm. (D) Nanog and Oct4 staining in e5.5 control and Nodal null embryos. (E) Nanog staining in e6.5 control and Nodal null BV^+ embryos. (F) Ap2γ staining in e6.5 control and Nodal null BV^+ embryos (G) Ap2γ staining in e7.5 control and Nodal null BV^+ embryos. (H) Blimp1 IHC in e7.5 control, EomesΔVE and EomesΔEpi embryos. Boxed areas indicated expanded panels on the right of the image. Scale bar = 200μm. (I) Eomes staining in mid-late streak stage control BV^+ embryos. All IF scale bars = 100μm.

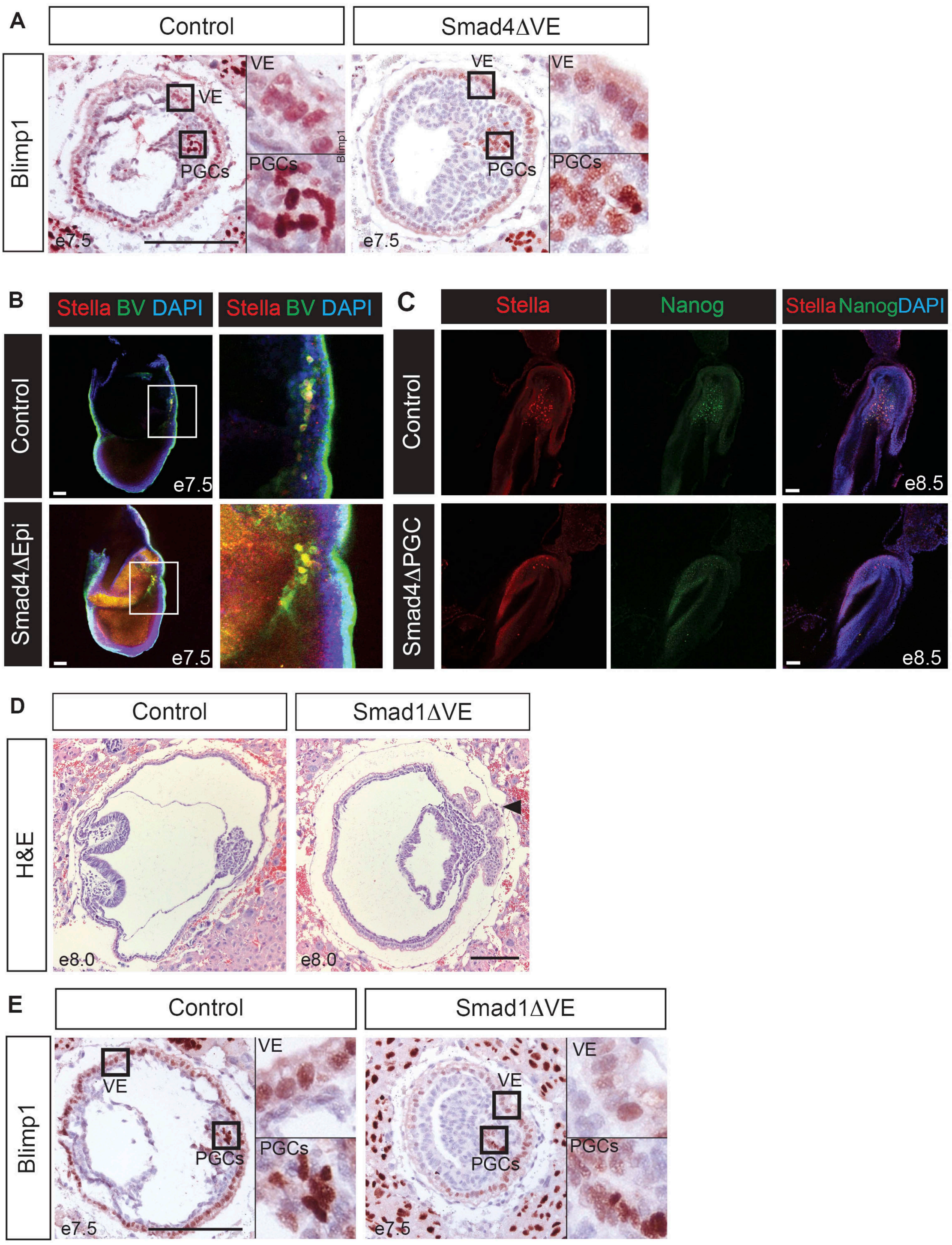

Supplementary figure 3: **Smad4 and Smad1 requirements for promoting PGC development.**

(A) Blimp1 IHC staining of e7.5 control and Smad4ΔVE embryo sections. Boxed areas indicated expanded panels on the right of the image. Scale bar = 200µm. (B) Stella expression in e7.5 control and Smad4ΔEpi BV^{+} embryos. (C) Optical sections of the posterior region of e8.5 control and Smad4ΔPGC embryos co-stained with Stella and Nanog antibodies, counterstained with DAPI. All IF scale bars = 100µm. (D) H&E staining of e8.0 control and Smad1ΔVE embryo sections. Arrowhead indicates ruffling of the posterior VE in Smad1ΔVE embryos. (E) Blimp1 IHC in control and Smad1ΔVE e7.5 embryo sections. Boxed areas indicated expanded panels on the right of the image. Scale bar = 200µm.

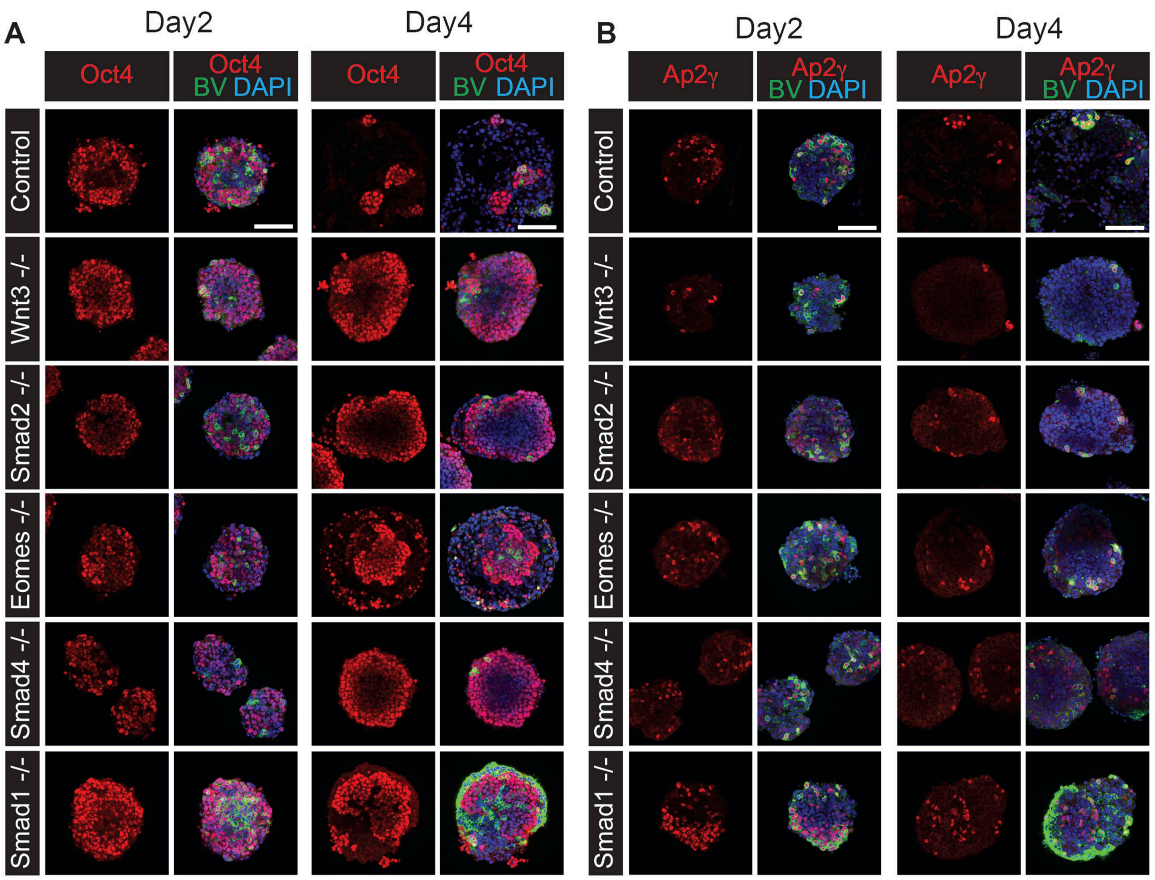

Supplementary figure 4: **PGC marker gene expression in wild type compared to Nodal, Bmp and Wnt signalling mutant PGCLC aggregates**.

(A) Oct4 staining of day 2 and day 4 PGCLC BV^+ aggregates of indicated genotypes, counterstained with DAPI. (B) Ap2γ staining of day 2 and day 4 PGCLC BV^+ aggregates of indicated genotypes, counterstained with DAPI. All scale bars = 100μm.

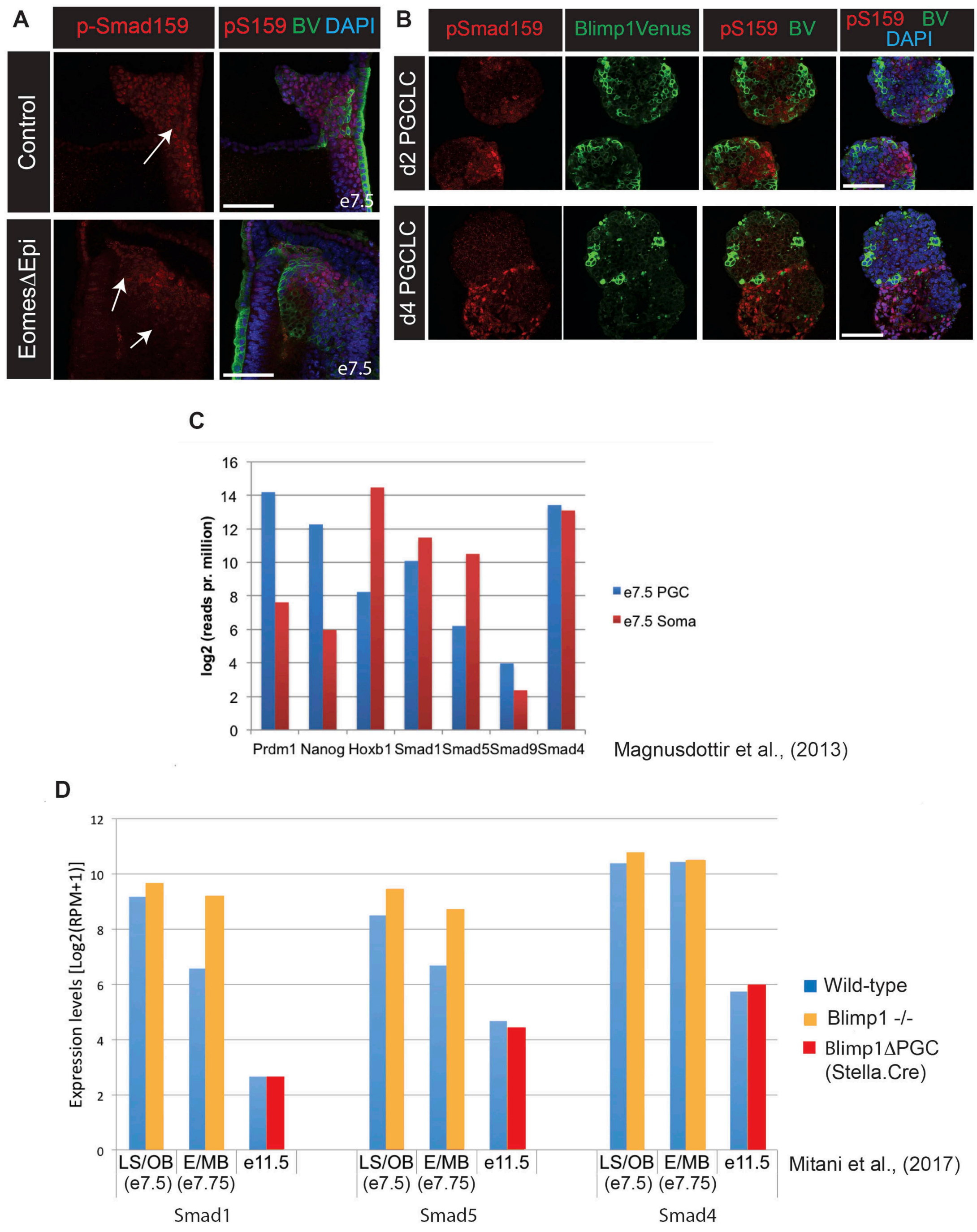

Supplementary figure 5: **PGCs selectively repress expression of Bmp/Smad signalling components.**

(A) p-Smad159 staining in e6.5 control and EomesΔEpi BV$^+$ embryos. Arrows indicate loss of p-Smad159 signal in the BV$^+$ cell population (B) p-Smad159 staining in BV+ wildtype d2 and d4 PGCLC aggregates. All IF scale bars = 100μm. (C) RNA-seq data showing expression levels [log2 (reads per million)] of *Smad1*, *Smad5, Smad9* and *Smad4* in e7.5 PGCs (blue) compared to somatic cells (red)[Ref 1]. Expression levels of *Prdm1* and *Nanog* (PGC markers) and Hoxb1 (somatic cell marker) are shown for comparison. (D) RNA-Seq data at indicated time-points showing expression [Log2(reads per million +1)] of *Smad1, Smad5* and *Smad4* in BV$^+$ PGC cells isolated from wild type (blue), Blimp1 null (orange) or embryos where Blimp1 has been deleted from specified PGCs using a Stella-Cre deletor strain (Blimp1ΔPGC) (red)[Ref 2]. LS/OB, late streak/no bud stage embryo; E/MB, early/mid bud stage embryos.

Supplementary table 1: *Characterization of Prdm1ΔVE mice*

A. Genotypes of offspring from conditional deletion of *Prdm1* from VE

Prdm1$^{-/+}$*; Ttr-Cre*$^{+}$ x *Prdm1*$^{CA/CA}$

Genotype	*Prdm1*$^{+/CA}$	*Prdm1*$^{+/\Delta VE}$	*Prdm1*$^{m/CA}$	*Prdm1*$^{m/\Delta VE}$
No of animals	2	3	3	3
Percentage	18.2	27.3	27.3	27.3
Expected %	25	25	25	25

B. Viability test of *Prdm1*ΔVE intercross

Prdm1$^{m/\Delta VE}$ x *Prdm1*$^{m/\Delta VE}$

No of animals	9

Supplementary table 2: *Mouse strains used in this study*

Embryo name	Genotype	Phenotype Reference	Additional transgene (Ref 3)
Smad2ΔVE	*Smad2*$^{CA/-}$; *Ttr-Cre*$^{+/-}$		*Blimp1-mVenus*
Smad2ΔEpi	*Smad2*$^{CA/-}$; *Sox2-Cre*$^{+/-}$	Ref 4	
EomesΔVE	*Eomes*$^{CA/-}$; *Ttr-Cre*$^{+/-}$	Ref 5	
EomesΔEpi	*Eomes*$^{CA/-}$; *Sox2-Cre*$^{+/-}$	Ref 6	*Blimp1-mVenus*
Smad1ΔVE	*Smad1*$^{CA/-}$; *Ttr-Cre*$^{+/-}$		
Smad1ΔEpi	*Smad1*$^{CA/-}$; *Sox2-Cre*$^{+/-}$		*Blimp1-mVenus*
Smad1 null	*Smad1*$^{-/-}$	Ref 7	*Blimp1-mVenus*
Smad4ΔVE	*Smad4*$^{CA/-}$; *Ttr-Cre*$^{+/-}$	Ref 8	
Smad4ΔEpi	*Smad4*$^{CA/-}$; *Sox2-Cre*$^{+/-}$	Ref 9	*Blimp1-mVenus*
Smad4ΔPGC	*Smad4*$^{CA/-}$; *Prdm1-Cre*$^{+/-}$		*Blimp1-mVenus*
Prdm1ΔVE	*Prdm1*$^{CA/-}$; *Ttr-Cre*$^{+/-}$		
Prdm1 null	*Prdm1*$^{-/-}$	Ref 10	*Blimp1-mVenus*
Nodal null	*Nodal*$^{-/-}$	Ref 11	*Blimp1-mVenus*

Sox2-Cre reference: Ref 12. Ttr-Cre reference: Ref 13.

Supplementary table 3: *Antibodies and datasets used in this study*

Antibodies	SOURCE	IDENTIFIER
Rabbit polyclonal anti-Nanog	Abcam	Cat# ab80892, RRID: AB_2150114 Lot: GR40243-12
Goat polyclonal anti-Sox2	R&D Systems	Cat# AF2018, RRID: AB_355110
Rabbit polyclonal anti-Stella	Santa Cruz	Cat# sc-67249, RRID: AB_1128372, Lot: H1809
Goat polyclonal anti-Stella	R&D Systems	Cat# AF2566, RRID: AB_2094147, Lot:UWS0108111
Goat polyclonal anti-Oct4	Santa Cruz	Cat# sc-8628, RRID: AB_653551, Lot: F1815
Rabbit polyclonal anti-Ap2γ	Santa Cruz	Cat# sc-8977, RRID: AB_2286995, Lot: G1112
Goat polyclonal anti-Brachyury (N-19)	Santa Cruz	Cat# sc-17743 RRID: AB_634980 Lot: A1614
Rat monoclonal anti-E-Cadherin	Sigma-Aldrich	Cat#U3254 RRID: AB_477600 Lot: 085K4798
Rabbit polyclonal anti-Eomes	Abcam	Cat#ab23345 RRID: AB_778267 Lot:GR306193-1
Rabbit monoclonal anti-phospho Smad1/5/9	Cell Signaling Technology	Cat# 13820 RRID: AB_2493181 Lot: D5810
Goat polyclonal anti-Otx2	R&D Systems	Cat# AF1979, RRID: AB_2157172, Lot: KNO0615111

Rat monoclonal anti-Blimp1	Santa Cruz	Cat# sc-130917, RRID: AB_2169704, Lot: G0913
Rabbit polyclonal anti-GFP Alexa Fluor 488	Invitrogen	Cat# A21311, RRID: AB_221477, Lot: 1891008
Chicken polyclonal anti-GFP	Abcam	Cat# Ab13970, RRID: AB_300798, Lot: GR305729-1
Goat anti-chicken Alexa Fluor 488	Invitrogen	Cat# A11039, RRID: AB_142924,
Donkey anti-rabbit Alexa Fluor 594	Invitrogen	Cat# A21207, RRID: AB_141637
Donkey anti-goat Alexa Fluor 594	Invitrogen	Cat# A11058, RRID: AB_142540
Donkey anti-rabbit Alexa 488	Molecular Probes	Cat# A21206, RRID: AB_141708
Donkey anti-rat 594	Molecular Probes	Cat# A21209 RRID: AB_2535795
Rabbit anti-rat IgG	Vector Laboratories	Cat# AI-4001 RRID: AB_2336209
Used datasets	SOURCE	IDENTIFIER
Transcription profiling by high throughput sequencing of BV+ primordial germ cells from e7.5 embryos and e7.5 somatic neighbors	Magnusdottir et al. 2013 (Ref 1)	E-MTAB-1178 [https://www.ebi.ac.uk/arrayexpress/experiments/E-MTAB-1178/]
Expression profiling by high throughput sequencing of LS/OB (e7.5) and E/MB (e7.75) BV+ primordial germ cells from Blimp1 KO and BV+ wild type control embryos as well as BV+ e11.5 primordial germ cells from Stella.Cre driven Blimp1 cKO embryonic gonads and BV+ wild type controls	Mitani et al. 2017 (Ref 2)	GSE91040 [https://www.ncbi.nlm.nih.gov/geo/query/acc.cgi?acc=GSE91040] (original data therein derived from GSE11128 and GSE74094)
Blimp1-EGFP ChIP-seq in murine d2 and d6 PGCLCs	Mitani et al. 2017 (Ref 2)	GSE91041 [https://www.ncbi.nlm.nih.gov/geo/query/acc.cgi?acc=GSE91041]

Supplementary table 4: *qRT-PCR primers used in this study*

Gene Name	Forward Primer Sequence	Reverse Primer Sequence	Annealing temperature	Product
Prdm1	GGCTCCACTACCCTTATCCTG	TCCTTTTGGAGGGATTGGAGTC	55°C	171bp
Tfap2c	GCCGGACGCCATGTTGTGGA	ACCCCGGTGTGCGAGAGAGG	55°C	162bp
Hoxb1	AGCCTACGACCTCCTCTCTG	GTGGTGAAGTTTGTGCGGAG	55°C	171bp
Gapdh	TGCACCACCAACTGCTTAGC	GGCATGGACTGTGGTCATGAG	55°C	87bp

References

1. Magnusdottir, E. *et al.* A tripartite transcription factor network regulates primordial germ cell specification in mice. *Nat Cell Biol* **15**, 905-915, doi:10.1038/ncb2798 (2013).
2. Mitani, T. *et al.* Principles for the regulation of multiple developmental pathways by a versatile transcriptional factor, BLIMP1. *Nucleic Acids Res* **45**, 12152-12169, doi:10.1093/nar/gkx798 (2017).
3. Ohinata, Y., Sano, M., Shigeta, M., Yamanaka, K. & Saitou, M. A comprehensive, non-invasive visualization of primordial germ cell development in mice by the Prdm1-mVenus and Dppa3-ECFP double transgenic reporter. *Reproduction* **136**, 503-514, doi:10.1530/REP-08-0053 (2008).
4. Vincent, S. D., Dunn, N. R., Hayashi, S., Norris, D. P. & Robertson, E. J. Cell fate decisions within the mouse organizer are governed by graded Nodal signals. *Genes Dev* **17**, 1646-1662, doi:10.1101/gad.1100503 (2003).
5. Nowotschin, S. *et al.* The T-box transcription factor Eomesodermin is essential for AVE induction in the mouse embryo. *Genes Dev* **27**, 997-1002, doi:10.1101/gad.215152.113 (2013).
6. Arnold, S. J., Hofmann, U. K., Bikoff, E. K. & Robertson, E. J. Pivotal roles for eomesodermin during axis formation, epithelium-to-mesenchyme transition and endoderm specification in the mouse. *Development* **135**, 501-511, doi:10.1242/dev.014357 (2008).
7. Tremblay, K. D., Dunn, N. R. & Robertson, E. J. Mouse embryos lacking Smad1 signals display defects in extra-embryonic tissues and germ cell formation. *Development* **128**, 3609-3621 (2001).
8. Li, C., Li, Y. P., Fu, X. Y. & Deng, C. X. Anterior visceral endoderm SMAD4 signaling specifies anterior embryonic patterning and head induction in mice. *Int J Biol Sci* **6**, 569-583 (2010).
9. Chu, G. C., Dunn, N. R., Anderson, D. C., Oxburgh, L. & Robertson, E. J. Differential requirements for Smad4 in TGFbeta-dependent patterning of the early mouse embryo. *Development* **131**, 3501-3512, doi:10.1242/dev.01248 (2004).
10. Vincent, S. D. *et al.* The zinc finger transcriptional repressor Blimp1/Prdm1 is dispensable for early axis formation but is required for specification of primordial germ cells in the mouse. *Development* **132**, 1315-1325, doi:10.1242/dev.01711 (2005).
11. Conlon, F. L., Barth, K. S. & Robertson, E. J. A novel retrovirally induced embryonic lethal mutation in the mouse: assessment of the developmental fate of embryonic stem cells homozygous for the 413.d proviral integration. *Development* **111**, 969-981 (1991).
12. Hayashi, S., Lewis, P., Pevny, L. & McMahon, A. P. Efficient gene modulation in mouse epiblast using a Sox2Cre transgenic mouse strain. *Mech Dev* **119 Suppl 1**, S97-S101 (2002).
13. Kwon, G. S. & Hadjantonakis, A. K. Transthyretin mouse transgenes direct RFP expression or Cre-mediated recombination throughout the visceral endoderm. *Genesis* **47**, 447-455, doi:10.1002/dvg.20522 (2009).
