# Supplementary material for: Genetic dissection of Nodal and Bmp signalling requirements during primordial germ cell development in mouse
Source: Nat Commun. 2019 Mar 6;10:1089. doi: 10.1038/s41467-019-09052-w (PMC6403387; doi:10.1038/s41467-019-09052-w)
Supplement: Supplementary file 3 — Reporting Summary [file 41467_2019_9052_MOESM3_ESM.pdf]

nature research

Corresponding author(s): Elizabeth J. Robertson

Last updated by author(s): Jan 28, 2019

Reporting Summary

Nature Research wishes to improve the reproducibility of the work that we publish. This form provides structure for consistency and transparency in reporting. For further information on Nature Research policies, see Authors & Referees and the Editorial Policy Checklist.

Statistics

For all statistical analyses, confirm that the following items are present in the figure legend, table legend, main text, or Methods section.

n/a	Confirmed	
☐	☒	The exact sample size (*n*) for each experimental group/condition, given as a discrete number and unit of measurement
☐	☒	A statement on whether measurements were taken from distinct samples or whether the same sample was measured repeatedly
☒	☐	The statistical test(s) used AND whether they are one- or two-sided<br>*Only common tests should be described solely by name; describe more complex techniques in the Methods section.*
☒	☐	A description of all covariates tested
☒	☐	A description of any assumptions or corrections, such as tests of normality and adjustment for multiple comparisons
☒	☐	A full description of the statistical parameters including central tendency (e.g. means) or other basic estimates (e.g. regression coefficient) AND variation (e.g. standard deviation) or associated estimates of uncertainty (e.g. confidence intervals)
☒	☐	For null hypothesis testing, the test statistic (e.g. *F*, *t*, *r*) with confidence intervals, effect sizes, degrees of freedom and *P* value noted<br>*Give P values as exact values whenever suitable.*
☒	☐	For Bayesian analysis, information on the choice of priors and Markov chain Monte Carlo settings
☒	☐	For hierarchical and complex designs, identification of the appropriate level for tests and full reporting of outcomes
☒	☐	Estimates of effect sizes (e.g. Cohen's *d*, Pearson's *r*), indicating how they were calculated

Our web collection on statistics for biologists contains articles on many of the points above.

Software and code

Policy information about availability of computer code

Data collection	N/A
Data analysis	N/A

For manuscripts utilizing custom algorithms or software that are central to the research but not yet described in published literature, software must be made available to editors/reviewers. We strongly encourage code deposition in a community repository (e.g. GitHub). See the Nature Research guidelines for submitting code & software for further information.

Data

Policy information about availability of data

All manuscripts must include a data availability statement. This statement should provide the following information, where applicable:

- Accession codes, unique identifiers, or web links for publicly available datasets
- A list of figures that have associated raw data
- A description of any restrictions on data availability

All relevant data are available from the authors. Publicly available source data in Figure 5 and Supplementary figure 5 were obtained from GSE91041 [https://www.ncbi.nlm.nih.gov/geo/query/acc.cgi?acc=GSE91041], GSE91040 [https://www.ncbi.nlm.nih.gov/geo/query/acc.cgi?acc=GSE91040] and E-MTAB-1178 [https://www.ebi.ac.uk/arrayexpress/experiments/E-MTAB-1178/].

October 2018

Field-specific reporting

Please select the one below that is the best fit for your research. If you are not sure, read the appropriate sections before making your selection.

☒ Life sciences ☐ Behavioural & social sciences ☐ Ecological, evolutionary & environmental sciences

For a reference copy of the document with all sections, see nature.com/documents/nr-reporting-summary-flat.pdf

Life sciences study design

All studies must disclose on these points even when the disclosure is negative.

Sample size	Multiple litters of each intercross were examined, with a minimum of n=3 mutant embryos stained and imaged.
Data exclusions	N/A
Replication	Multiple litters of each intercross were examined, with a minimum of n=3 mutant embryos stained and imaged.
Randomization	Embryos were imaged and subsequently retrospectively genotyped to exclude any bias.
Blinding	Embryos were imaged and subsequently retrospectively genotyped to exclude any bias.

Reporting for specific materials, systems and methods

We require information from authors about some types of materials, experimental systems and methods used in many studies. Here, indicate whether each material, system or method listed is relevant to your study. If you are not sure if a list item applies to your research, read the appropriate section before selecting a response.

Materials & experimental systems

n/a	Involved in the study	
☐	☒	Antibodies
☐	☒	Eukaryotic cell lines
☒	☐	Palaeontology
☐	☒	Animals and other organisms
☒	☐	Human research participants
☒	☐	Clinical data

Methods

n/a	Involved in the study	
☒	☐	ChIP-seq
☒	☐	Flow cytometry
☒	☐	MRI-based neuroimaging

Antibodies

Antibodies used

Rabbit polyclonal anti-Nanog: Abcam, Cat# ab80892, RRID: AB_2150114, Lot: GR40243-12;
Goat polyclonal anti-Sox2, R&D Systems, Cat# AF2018, RRID: AB_355110;
Rabbit polyclonal anti-Stella, Santa Cruz, Cat# sc-67249, RRID: AB_1128372,
Lot: H1809;
Goat polyclonal anti-Stella, R&D Systems, Cat# AF2566,
RRID: AB_2094147, Lot:UWS0108111;
Goat polyclonal anti-Oct4, Santa Cruz, Cat# sc-8628, RRID: AB_653551, Lot: F1815;
Rabbit polyclonal anti-Ap2γ, Santa Cruz, Cat# sc-8977, RRID: AB_2286995, Lot: G1112;
Goat polyclonal anti-Brachyury (N-19), Santa Cruz, Cat# sc-17743 RRID: AB_634980, Lot: A1614;
Rat monoclonal anti-E-Cadherin, Sigma-Aldrich, Cat#U3254, RRID: AB_477600, Lot: 085K4798;
Rabbit polyclonal anti-Eomes, Abcam, Cat#ab23345 RRID: AB_778267, Lot:GR306193-1;
Rabbit monoclonal anti-phospho Smad1/5/9, Cell Signaling Technology, Cat# 13820
RRID: AB_2493181, Lot: D5810;
Goat polyclonal anti-Otx2, R&D Systems, Cat# AF1979, RRID: AB_2157172, Lot: KNO0615111;
Rat monoclonal anti-Blimp1, Santa Cruz, Cat# sc-130917, RRID: AB_2169704,
Lot: G0913;
Rabbit polyclonal anti-GFP Alexa Fluor 488; Invitrogen; Cat# A21311, RRID: AB_221477, Lot: 1891008;
Chicken polyclonal anti-GFP, Abcam, Cat# Ab13970, RRID: AB_300798,
Lot: GR305729-1;
Goat anti-chicken Alexa Fluor 488, Invitrogen, Cat# A11039, RRID: AB_142924;
Donkey anti-rabbit Alexa Fluor 594, Invitrogen, Cat# A21207, RRID: AB_141637;
Donkey anti-goat Alexa Fluor 594, Invitrogen, Cat# A11058, RRID: AB_142540;
Donkey anti-rabbit Alexa 488, Molecular Probes, Cat# A21206, RRID: AB_141708;
Donkey anti-rat 594, Molecular Probes, Cat# A21209 RRID: AB_2535795;
Rabbit anti-rat IgG, Vector Laboratories, Cat# AI-4001 RRID: AB_2336209

Validation	The staining patterns for each primary antibody used were validated in wild type embryos and compared to published antibody/WISH data.

Eukaryotic cell lines

Policy information about cell lines

Cell line source(s)	Embryionic stem cell lines were derived from blastocysts obtained from intercrosses of Wnt3+/- (Ref 23), Smad1+/- (Ref 15), Smad2+/- (Ref 25), Smad4+/- (Ref 39) and Eomes+/- (Ref 36) mice harbouring the Prdm1-mVenus BAC transgene (Ref 32).
Authentication	Embryonic stem cell lines were genotyped with allele specific primers as described in the original publications (see above)
Mycoplasma contamination	N/A
Commonly misidentified lines (See ICLAC register)	N/A

Animals and other organisms

Policy information about studies involving animals; ARRIVE guidelines recommended for reporting animal research

Laboratory animals	Mus musculus, strains as detailed in Supplementary Table 2. Sexually mature males and females (6 weeks +) of the indicated genotypes were intercrossed and pregnant females at the indicated gestational stages were sacrificed for embryo recovery.
Wild animals	N/A
Field-collected samples	N/A
Ethics oversight	All animal experiments were performed in accordance with Home Office (UK) regulations and approved by the University of Oxford local Ethical Committee.

Note that full information on the approval of the study protocol must also be provided in the manuscript.
